# Public preferences for communicating personal genomic risk information: a focus group study

**DOI:** 10.1111/hex.12406

**Published:** 2015-09-01

**Authors:** Amelia K. Smit, Louise A. Keogh, Jolyn Hersch, Ainsley J. Newson, Phyllis Butow, Gabrielle Williams, Anne E. Cust

**Affiliations:** ^1^ Cancer Epidemiology and Services ResearchSydney School of Public HealthThe University of SydneySydneyNSWAustralia; ^2^ Centre for Women's Health, Gender and SocietyThe University of MelbourneMelbourneVic.Australia; ^3^ Screening and Test Evaluation ProgramSydney School of Public HealthThe University of SydneySydneyNSWAustralia; ^4^ Centre for Values, Ethics and the Law in MedicineSydney School of Public HealthThe University of SydneySydneyNSWAustralia; ^5^ Centre for Medical Psychology and Evidence‐based Decision‐makingSchool of PsychologyThe University of SydneySydneyNSWAustralia; ^6^ Centre for Genetics EducationNSW Government Department of HealthSydneyNSWAustralia

**Keywords:** communication, genetic, genomic, melanoma, public preferences, risk

## Abstract

**Background:**

Personalized genomic risk information has the potential to motivate behaviour change and promote population health, but the success of this will depend upon effective risk communication strategies.

**Objective:**

To determine preferences for different graphical and written risk communication formats, and the delivery of genomic risk information including the mode of communication and the role of health professionals.

**Design:**

Focus groups, transcribed and analysed thematically.

**Participants:**

Thirty‐four participants from the public.

**Methods:**

Participants were provided with, and invited to discuss, a hypothetical scenario giving an individual's personalized genomic risk of melanoma displayed in several graphical formats.

**Results:**

Participants preferred risk formats that were familiar and easy to understand, such as a ‘double pie chart’ and ‘100 person diagram’ (pictograph). The 100 person diagram was considered persuasive because it humanized and personalized the risk information. People described the pie chart format as resembling bank data and food (such as cake and pizza). Participants thought that email, web‐based platforms and postal mail were viable options for communicating genomic risk information. However, they felt that it was important that a health professional (either a genetic counsellor or ‘informed’ general practitioner) be available for discussion at the time of receiving the risk information, to minimize potential negative emotional responses and misunderstanding. Face‐to‐face or telephone delivery was preferred for delivery of high‐risk results.

**Conclusions:**

These public preferences for communication strategies for genomic risk information will help to guide translation of genome‐based knowledge into improved population health.

## Introduction

Advances in genomic technologies and improved knowledge of the role of genomics in common diseases now make it feasible and potentially cost‐effective to use genomic information for risk stratification and interventions aimed at disease prevention on a population scale.^1^, ^2^ Whilst ‘genetic risk’ focuses on rare mutations in single genes, ‘genomic risk’ refers to a person's risk of disease based on common variation in a number of genes. The potential of personalized genomic risk information to motivate behaviour change and promote health is a burgeoning area of research that depends at least in part on the identification of effective risk communication strategies.^3^ Therefore, accessible and understandable formats for the communication of personalized genomic risk are vital.^4^ Studies of risk communication formats have demonstrated that probabilistic information is difficult to convey and that individuals do not easily grasp concepts of risk.^5^, ^6^ Such studies have explored different delivery models and established that factors such as literacy and numeracy influence recipients' understanding of risk.^5^, ^7^, ^8^

However, most studies of communicating genetic risk to date have concentrated on delivering information about rare, single gene mutations that carry a high risk of disease,^9^ mostly among families with a strong family history.^10^ Most common diseases such as cancer have complex multifactorial causes and are much more frequently influenced by multiple genetic and environmental factors than by single gene mutations.^11^ Compared to disease risk based on single genes, genomic risk is based on small effects of variants in multiple genes, which in combination can have a large influence on risk.^11^ Genomic variants are common in the population compared to high‐risk mutations and thus make a significant contribution to disease burden. The patterns of inheritance for genomic risk are also more complex than for single gene mutations as these are based on multiple probabilities and are not easily visualized or explained using a family history. Although a number of risk presentation formats have been evaluated in the literature, few have displayed genomic risk information and been tested among the wider public.^12^

Individual preferences for the delivery of genomic risk information to the wider population therefore remain relatively unexplored. The acceptability of different modes of communicating genetic risk information, such as via online platforms, email, telephone or face‐to‐face, and the role of health professionals in the risk communication process are becoming increasingly relevant because genetic counselling providers are already experiencing strain and beginning to streamline their practices.^13^ This is partly due to the vast amounts of genomic data being generated from emerging genomic technologies.^14^

We used focus group discussions to determine preferred strategies for communicating personal genomic risk of melanoma to the public. Melanoma is the most serious form of skin cancer, and Australia has the world's highest incidence of this disease.^15^ Excessive sun exposure is a strong risk factor for melanoma, making it a highly preventable disease^16^, ^17^; however, skin cancer prevention and detection behaviours remain suboptimal for most Australians.^18^ Genomic variants have also been shown to be strong predictors of melanoma risk.^19^, ^20^

As the public are generally not familiar with genomic risk information,^21^ we explored participants' preferences using focus group methodology. Focus groups are particularly suited to new areas of research as the interactive nature of discussions stimulates participants' thoughts about topics they may not normally discuss.^22^, ^23^ Furthermore, interaction with the researcher has less of an influence on discussion in a focus group than in an interview.^24^

We presented focus group participants with a hypothetical scenario that displayed an individual's personalized genomic risk of melanoma using different graphical formats. Specific objectives of the study were to determine preferences for:

1. different graphical and written formats for risk communication; and
2. the delivery of genomic risk information, including the mode of communication and the role of health professionals in the communication process.

## Methods

### Participant recruitment

Participants for this study were recruited via the ‘Join a research study’ database managed by the Cancer Council New South Wales (NSW), Australia. Members of this database comprise a mix of demographics including people with cancer, relatives, friends and the wider public. All have given consent to be contacted by researchers carrying out ethically approved research studies related to cancer. Ethics approval was obtained from The University of Sydney. We received contact details from the ‘Join a research study’ database for individuals who met the participant criteria required for our study: 18 or more years of age and no personal history of melanoma. To establish diversity as well as geographical representativeness, invitation letters were sent to central, western, northern and southern locations across Sydney, and the focus groups 1, 2, 3 and 4 were offered in these locations, respectively.

Packs including an invitation letter, participant information sheet, consent form, participation card and reply paid envelope were sent via postal mail to 200 individuals; six were returned due to an incorrect address. Once a participant returned their consent form, we contacted them to discuss the study further and to allocate them to the focus group session most convenient for them. In response to the invitation mail‐out, 43 (22%) gave their consent to participate in a focus group discussion and an additional 25 (13%) gave their consent to participate in a phone interview if needed. Thirty‐four participants ultimately attended the four focus groups, which were made up of 5, 12, 8 and 9 participants, respectively. A $50 gift voucher was given to each participant to compensate them for their travel expenses and time.

### Focus group discussion

**Figure 1:**
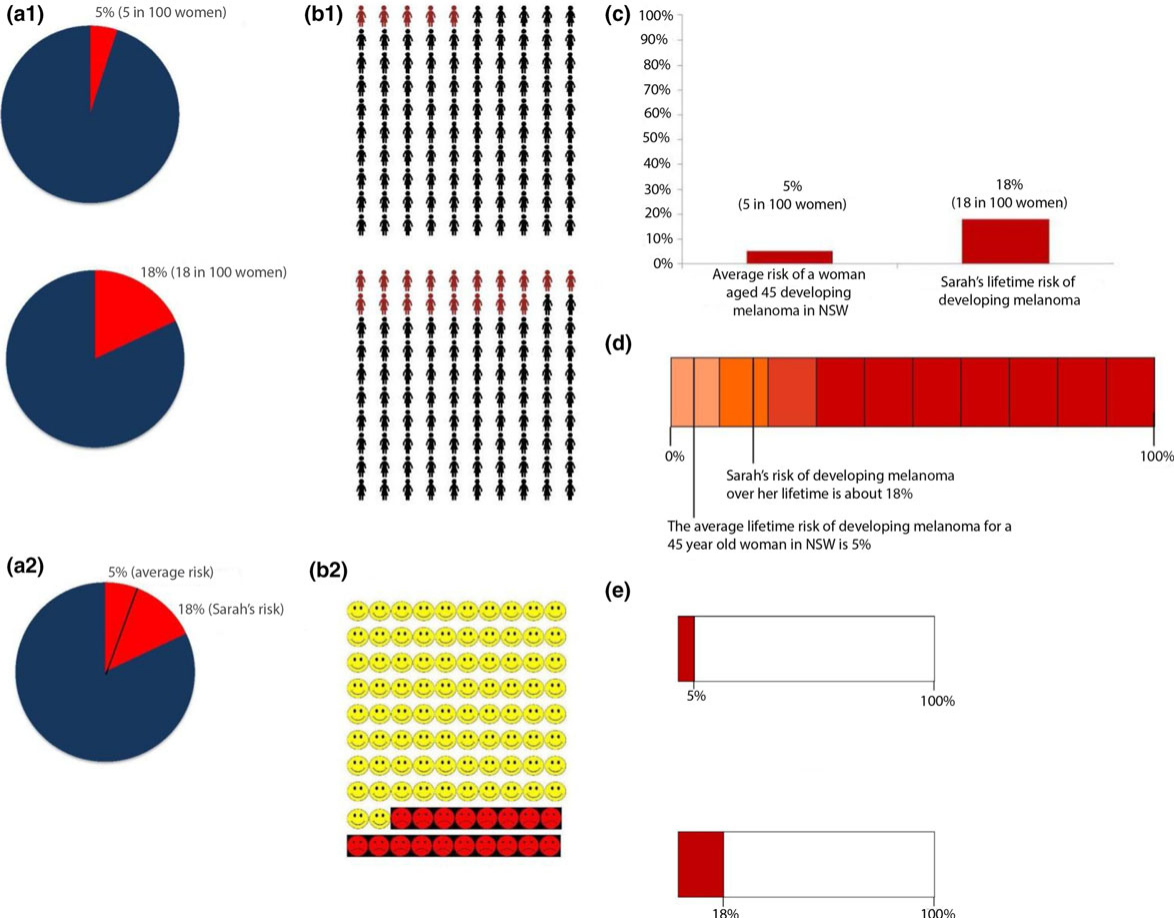
The different risk presentations discussed in the focus groups. a1 is the double pie chart, a2 is the merged pie chart, b1 is the 100 person diagram (pictograph), b2 is the 100 faces diagram, c is the bar graph, d is the scale diagram and e is the box plot. A scenario describing ‘Sarah’ was given to participants before presenting the different risk presentations. The text shown below accompanied each risk presentation format, and every format was presented on a separate page. For the risk formats where two graphs were shown on the same page (e.g. a1, b1, e), the graph showing average risk was shown under the ‘Average Risk’ text and the graph showing Sarah's risk was shown under the ‘Sarah's Risk’ text. Accompanying text for each graph: *Average risk*: For a 45‐year‐old woman in NSW, the average lifetime risk of developing melanoma is 5%. This means that, on average, of 100 women living in NSW who are the same age as Sarah, 5 will develop melanoma over their lifetime. *Sarah's risk*: Based on Sarah's age and her genetic risk information, her lifetime risk of developing melanoma is about 18%. This means that out of every 100 women with the same age and genetic risk as Sarah, 18 women are likely to develop melanoma over their lifetime. Sarah is about 3.6 times more at risk of developing melanoma from now until the age of 85, than other women her age in NSW. [Colour figure can be viewed at wileyonlinelibrary.com]

Focus groups were conducted by an experienced facilitator. They comprised two parts in a single two‐hour session, including a 15‐min break. Discussion was conversational, guided by a semi‐structured focus group Discussion Guide with a theme list and prompts. The Discussion Guide is shown in Appendix S1 (online supplementary material). We used the word ‘genetic’ rather than ‘genomic’ to facilitate understanding among the public. In the first half of the session, participants discussed personalized (hypothetical) genetic risk information that was presented in several different formats (Fig. 1). We based our formats on frequently used risk presentations in previous studies of disease risk communication. We followed strategies for presenting risk information proposed by Lautenbach *et al*.^12^ and selected different visual representations of disease risk appropriate for a range of numeracy levels^5^, ^25^, ^26^, ^27^, ^28^, ^29^ and accompanying text that describes the risk in relative and absolute terms. ‘Genetic make‐up’ was described by facilitators at the beginning of the discussion (Appendix S1), and participants were invited to discuss their understanding and to raise any questions regarding risk as presented in Fig. 1.

The risk information showed an 18% lifetime genetic risk of melanoma for ‘Sarah’, a hypothetical 45‐year‐old woman living in New South Wales, Australia. The lifetime risk calculation was based on her (hypothetical) genomic variation in 18 different genes, her age, sex and the State in which she lived (as melanoma incidence varies strongly by age, sex and ambient solar ultraviolet radiation). The information also compared her risk to the average woman of her age living in the same place.

Participants were invited to comment on the different risk presentation formats and to make recommendations that would improve the presentation and thereby understanding of this information. After the group discussions, participants were asked to rank the formats on a paper form, according to their personal preference with 1 indicating their favourite format and 7 indicating their least favourite. In the second half of the session, participants were asked about preferences for different modes of communication of genetic risk information such as postal mail, online, email, telephone and face‐to‐face. Preferences for the role of health professionals, particularly general practitioners (GPs) and genetic counsellors, in the risk communication process were also discussed. To facilitate discussion about health professionals, the role of genetic counsellors in the New South Wales health‐care system was explained to participants.

### Data capture, coding, and analysis of qualitative data

The focus groups were audio recorded, transcribed verbatim by a professional transcription service, and analysed thematically. NVivo qualitative data analysis software (QSR International Pty Ltd. Version 10) supported the coding process. Initially, a working coding framework was developed, which was structured according to research questions and the Discussion Guide. Through an iterative process of reading and re‐reading the transcripts, additional themes and subcodes were identified and added to the coding framework. Inductive reasoning was relied on to allocate phrases, words and paragraphs to both the top‐level codes and subcodes. The data within each theme were then further analysed to identify variations or patterns present. Coding was performed by AKS and AEC. Discrepancies were discussed between AKS, AEC and LAK, and agreement was reached by consensus.

## Results

The average age of participants was 56 years (range 19‐83 years) and almost three quarters of participants held a university level qualification (Table 1). One in five (21%) participants reported that they had been diagnosed with non‐melanoma skin cancer (basal cell carcinoma or squamous cell carcinoma); a proportion consistent with Australian population data that report two‐thirds of Australians have experienced at least one non‐melanoma skin cancer before the age of 70 years.^30^ One in seven (15%) participants reported that they had an immediate family member who had been diagnosed with melanoma. Age and gender distribution were fairly similar across the four focus groups.

**Table 1:** Demographic characteristics of participants

Characteristics	Number (%) *n* = 34^a^
Gender	
Males	9 (27)
Females	24 (73)
Age (years)	
18‐45	10 (30)
46‐65	12 (36)
66‐85	11 (33)
Education	
Some high school	0 (0)
High School	4 (12)
Technical college certificate or diploma	5 (15)
University diploma or degree	24 (73)
Country of birth	
Australia	24 (73)
Other	9 (27)
Ethnic background	
Caucasian/white	26 (79)
South Asian	3 (9)
Middle Eastern	1 (3)
Pacific Islander	1 (3)
Other	2 (6)
Hair colour at age 18	
Red	2 (6)
Blonde	3 (9)
Light or mouse brown	12 (36)
Dark brown	13 (39)
Black	3 (9)
Eye colour	
Black/brown	15 (45)
Blue or grey	12 (36)
Green or hazel	6 (18)

a One participant attended a focus group but did not complete a questionnaire.

### Preferences for different risk presentation formats

Participants generally preferred the formats that they believed clearly communicated and visually reflected the difference between Sarah's risk and the average lifetime melanoma risk and that featured the fewest numbers. Based on overall individual preferences (Table 2), the double pie chart was ranked first (Fig. 1a1) and a pictograph, which we refer to as the ‘100 person diagram’, was ranked second (Fig. 1b1). The bar graph was ranked third (Fig. 1c), the scale diagram (Fig. 1d) fourth and the box plot (Fig. 1e) fifth. Participants in focus groups 1 and 2 made several suggestions for the risk presentation formats, and taking these into consideration, we included two additional formats in focus groups 3 and 4: a 100 ‘face’ (emoticon) diagram (Fig. 1b2) and a ‘merged’ pie chart (Fig. 1a2). Participants in these latter focus groups had mixed views of the 100 faces diagram and the merged pie chart. Overall, these participants preferred the 100 person diagram and double pie chart.

**Table 2:** Participant preferences for different risk presentation formats

Preferences	Group 1	Group 2	Group 3	Group 4	Overall Ranking^a^
1	100 person diagram	100 person diagram	Bar graph	Merged pie chart	Double pie chart
2	Double pie chart	Double pie chart	Double pie chart	Scale diagram	100 person diagram
3	Bar graph	Bar graph	Box plot	Double pie chart	Bar graph
4	Box plot	Box plot	100 person diagram	100 person diagram	Box plot
5	Scale diagram	Scale diagram	Merged pie chart	Bar graph	Scale diagram
6			Scale diagram	Box plot	
7			100 faces diagram	100 faces diagram	

a The overall ranking displays the preferences for the five risk formats that were presented in all four focus groups.

#### Preference 1: Double Pie Chart

Participants described the double pie chart format as similar to ‘bank data’ and other familiar items:

Female (focus group 1) I like pie graphs, I always think they're very easy to visualize because people are so used to cutting up cake and pizza.

One participant stated they disliked pie charts. Other participants believed the pie charts were effective because they could be understood separately from the text. Generally, participants criticized formats in which they believed the text must be read to understand the graph. Some participants mentioned that they were not comfortable with numbers and said the absence of many numbers in the pie chart made it easier to understand. Participants observed that they found it easy to understand Sarah's risk in relation to the average due to the labelling of the pie charts: ‘5% (5 in 100 women)’. Additionally, participants noted that the difference in percentages was more obvious in the pie charts than in the other formats, and thus, the pie charts made Sarah's risk in relation to the average clearer.

#### Preference 2: 100 Person Diagram

Participants stated that the 100 person diagram (Fig. 1b1) was a familiar format which reminded them of health and bank information, and thus was ‘appropriate for the general public’. Whilst some criticized the format as ‘too much at once’ most found it easy to understand. This was the only format that participants thought humanized or personalized the risk information. Furthermore, participants labelled it as the ‘most persuasive’ and observed that this format clearly displayed Sarah's higher risk compared with the average risk estimate:

Female (focus group 4) Her increase of risk, it stands out there, oh my goodness me, I'm one of those women, that's a lot.

Some participants expressed that the 100 person diagram was ‘more pessimistic’ than the other examples and one participant stated:

Male (focus group 1) I reckon if you were Sarah you'd be worried.

#### Criticisms of alternative formats

Participants criticized those formats that they perceived as being either too simplistic or too scientific. The 100 ‘face’ diagram (presented to focus groups 3 and 4) was described as ‘emotive’ and ‘childish’. It made some participants think about death:

Female (focus group 4) Is it giving the information out to Sarah that she's going to die because it's got sad faces?

Male (focus group 4) That's what I was thinking, yeah.

Participants said they would be worried if they received the bar graph (Fig. 1c) and believed that it was an intimidating representation of the risk estimate, which reminded them of medical, research and financial reports. The box plot (Fig. 1e) was criticized for being ‘misleading’ as participants believed that Sarah's risk did not appear ‘serious’ enough. Participants stated in relation to this format:

Female (focus group 2) (The box plot) it's too bland, it doesn't make you think about it or anything.

Female (focus group 2) Yeah, not very strong.

The scale diagram (Fig. 1d) was described as ‘scary’, and the participants found it difficult to interpret. Some participants struggled to identify Sarah's risk in the ‘merged’ pie chart (Fig. 1a2), but others described it as ‘easy to read and memorable’.

#### Relative risk vs. absolute risk

There were different preferences for the presentation of risk estimates as relative risk or absolute risk estimates. One participant disliked the relative risk figure as it suggested to them that they were being compared to others. Another participant disagreed and maintained that it is important to emphasize peer comparison. Similarly, a participant proposed that the relative figure ‘goes straight to the point’. Others proposed that both relative and absolute estimates should be included as people are likely to have different preferences.

### Understanding risk

Lifetime risk and melanoma genetic risk were new concepts for many focus group participants.

Female (focus group 4) I think the most important thing [when communicating genetic risk information] is actually to let people know there is a genetic risk. (…) Because a lot of us came here not knowing.

Some participants asked why the age of 85 was used as the upper limit and had not understood that it was referring to their ‘residual’ lifetime risk, that is the risk from their current age until age 85, not their risk from birth to age 85 years. Some participants suggested rephrasing this to ‘*remaining* lifetime risk’ rather than simply ‘lifetime risk’ to emphasize this point. Several participants asked whether the absolute risk can be changed or will change over time and whether or not genetic risk can be changed. The Discussion Guide and risk information included simple information about common gene changes and inheritance, but did not specifically describe how genomic risk was calculated, to avoid overwhelming participants with information. However, many participants asked questions about this, including how a person's risk related to geographic location, age, phenotype (e.g. skin colour, moles), and the extent to which the environment and genetic factors influence the development of melanoma. The facilitator briefly responded to these questions including describing the number of genes involved in the melanoma risk calculation.

Some participants found it difficult to identify whether or not 18% signified a high risk. As the scale goes to 100 and 18% is a low number, participants raised the possibility that the absolute risk could be misinterpreted as low risk and therefore be ‘a bit deceiving’. They suggested including qualitative risk categories (e.g. low, average, high) in addition to the estimates of absolute and relative risk to minimize this potential misunderstanding. Participants also noted that they would want to know what the highest risk estimate could theoretically be:

Female (focus group 3) [The risk estimate needs] something that qualifies it against what would be considered high risk, so that you can actually put it in context I suppose.

### Delivery of information

#### Mode of communication

Participants acknowledged that the ability to select a mode of communication for receiving genetic risk information is important as people are likely to have different preferences. Pros and cons were identified for different mediums including email, online, postal mail, telephone and face‐to‐face communication. Some participants indicated that younger people may prefer online communication as they are more accustomed to using the internet. Participants provided contrasting examples of older people becoming ‘disenfranchised’ but also ‘more tech savvy than teens’. Participants suggested that online communication is a ‘common approach to tests’ with which people are familiar. Conversely, some participants were concerned that delivering risk information online or via email is impersonal. A number of participants viewed postal mail as more personal and preferred to receive risk information in the form of a hard copy. They noted that a hard copy would make it easier for them to discuss their results with friends, family and other health professionals. It was suggested that older people may generally prefer to receive the information in a hard copy format.

Genetic risk communication via a written‐only medium (of any kind) was identified by some participants as potentially distressing due to the possibility of self‐diagnosis, misunderstanding and a negative emotional reaction:

Female (focus group 1) I don't ever think you should tell people risk things online. I think because (…) you don't know what their emotional situation might be.

Participants believed that if risk information was communicated via a written medium, it would be important that the recipients receive it at a time when they are able to easily reach out and contact someone for appropriate support, that is not on a Friday afternoon.

#### The role of health professionals in the communication process

Several participants proposed that if an individual's risk level was high and therefore more likely to upset them, it may be more appropriate for them to receive their results from a health professional. There was general agreement among participants that this would be beneficial:

Female (focus group 4) I think person to person is good because I have self‐diagnosed on the computer and boy, you can have yourself dead.

Participants noted several benefits of face‐to‐face communication with a health professional, such as limiting the possibility of misinterpretation, providing emotional and psychological support, allowing for questions to be asked immediately and avoiding (possibly distressing) self‐diagnosis. Participants pointed out that it may be difficult for people who live in rural areas to see a health professional – a significant consideration in a country like Australia, which has a relatively small and widely dispersed population. Face time, Skype and the telephone were suggested as alternatives, which are becoming more common to communicate genetic risk information.^31^

Of the different types of health professionals, participants preferred to receive genetic information from either GPs or genetic counsellors. They emphasized that people are likely to have different preferences; therefore, they should be able to choose from whom they receive their risk information. Some participants believed that receiving genetic risk information from a GP would be beneficial as a GP represents a familiar mode of receiving test results, and may be better able to place the test results in context and advise ways of reducing risk than a genetic counsellor:

Female (focus group 3) If a genetic counsellor would be better placed to explain that risk, then I would say, yes I would be interested, but if what this is about is building that genetic risk into your lifestyle risk, then yeah, just go straight to your GP.

Limitations of receiving risk information from a GP included the belief that GPs may not know much about the risk information.

Female (focus group 4) I'd prefer the genetic counsellor.

Female (focus group 4) Definitely the specialist because a lot of GPs, I'm sorry, I hope there's no doctors here, but they're quite ignorant with a lot of things, I've found.

Whilst there was some uncertainty about the role of genetic counsellors, several participants believed that a genetic counsellor would be better able to understand and explain the risk information than a GP, and that they could provide support for people who may be ‘frightened’ or misunderstand their results. Participants also mentioned that they would only want to see a genetic counsellor if they were at high risk, otherwise they would consider it a waste of time.

## Discussion

As genomic information becomes more widespread, information to guide the risk communication process among the general public is essential for translating genome‐based knowledge into improved population health. Our study findings provide new insights into general public preferences for the communication of personalized genomic risk information, including the type of graphical and written formats, the mode of communication and the role of health professionals in the communication process.

Research on risk communication has demonstrated that preferences for risk formats are likely to vary and there is no consensus on how best to present personalized risk information.^29^ Therefore, we included a range of risk formats. Participant preferences reveal that the visual representation of genomic risk impacts emotional responses and associations with the information. Simplistic or ‘childish’ formats such as the 100 face diagram not only trivialized the information in the eyes of the participants in focus groups 3 and 4 but it also reminded them of death. On the other hand, more complex formats elicited the most references to emotions such as worry and fear as participants struggled to understand them.

The preferred graphical formats were the double pie chart and the 100 person diagram. Most participants found these formats easy to understand and had been exposed to them previously, for example when receiving financial information or other heath data. They also commented that these two formats clearly portrayed Sarah's higher risk in relation to the average risk. In a study of the impact of graphical presentation on health‐related knowledge and treatment choices by Hawley *et al*., the pie chart resulted in mixed responses. Hawley *et al*.^27^ found that the pie chart was least trustworthy and scientific according to participants. However, they also found that lower numeracy participants gained the most knowledge from the pie chart, followed by the pictograph. Participants in our study ranked the formats based on their personal preference and according to how well they could understand each visual representation. In our study, participants generally disliked formats they believed were ‘scientific’ and, similar to Hawley *et al*.'s findings, indicated that the pie chart and the 100 person diagram were the easiest to understand.

Participants noted that the 100 person diagram was persuasive and that it humanized and personalized the risk information and enabled them to visualize the risk estimate. The ‘personalization’ of genetic risk information, according to social and behavioural theory, is thought to be a more powerful motivator of healthy behaviour change than standard prevention approaches.^32^ Pictographs such as the 100 person diagram have been found to be the most effective method for communicating percentages and reducing the influence of anecdotal information on recipients' interpretation of risk.^12^, ^33^ Pictographs are also recommended for people with low literacy levels.^28^ Furthermore, it has been demonstrated that the framing of numerical risks in multiple ways, such as using graphics and frequency statements (e.g. of 100 women living in NSW who are the same age as you, 5 will develop melanoma over their remaining lifetime), aids recipients' understanding of risk.^12^ Participants in this study believed that the multiple ways of framing the numerical risks were helpful in understanding, which demonstrates that personalized genomic risk can be understood by individuals using existing standard risk communication formats.

Some participants experienced difficulty in understanding what ‘lifetime risk’ meant and exactly how the risk estimate was calculated. Given the complexity of calculating genomic risk information, we were originally uncertain of the level of detail participants would want to know. However, participants clearly wanted further detail about how different factors influence the risk calculation; thus, we suggest that this further detail should be presented alongside genomic risk information. Participants also recognized that the risk information could potentially be misunderstood or have a negative emotional impact on the recipient, particularly if the result revealed a high risk. There was strong support for having access to a GP or a genetic counsellor or health professional with appropriate training and expertise at the time of receiving genetic risk information. It was also proposed that if the risk result was ‘high’ then a health professional should deliver this information to the recipient either face‐to‐face or over the telephone. Telephone communication was described as a feasible mode of contact to discuss genetic risk information with a health professional.

The findings from our study suggest that email, web‐based and postal mail are all viable options for communicating genetic risk information, but that the ability to contact a health professional to discuss the information should be available at the time of receiving the information. For people at high genetic risk, it was considered that the delivery of the risk information should initially occur via a health professional either face‐to‐face or by telephone. Many participants were unsure about the role of a genetic counsellor, and these participants tended to prefer the option of receiving or discussing their risk results with their GP. Others who understood that genetic counsellors specialize in the communication of risk believed that a genetic counsellor was better placed to answer complex questions and deliver their results.

Other studies have also found that the ability to elect a mode of communication is important and that telephone delivery of genetic risk results is considered appropriate.^34^ A review of studies regarding communication of clinical research results^35^ found that participants often prefer to receive research results via a written format, with contact information provided, rather than attending an appointment face‐to‐face. In our study, participants considered online communication to be an increasingly common and acceptable mode of receiving medical information. Generally, younger people were identified as preferring online communication. A study on computer‐based cancer risk communication found that engagement and interactivity, facilitated by online platforms, aids understanding of disease risk and increases the likelihood of behaviour change to reduce risk.^36^ Email and online platforms are potential modes of risk communication that require further investigation. Interestingly, participants did not express any concerns about confidentiality in the delivery of the risk information.

Novel aspects of our study include a focus of genomic risk information, obtaining preferences from the wider public, and addressing both the format and delivery of the risk information. We have interpreted our findings in the context of the existing risk communication literature including among people with low literacy levels. The limitations of this study lie in participants' higher‐than‐average education levels and interest in cancer research, as their preferences for communication of genomic risk information may differ from other members of the public. Focus group methodology is limited by the possibility that assertive participants may contribute more or dominate the discussion and those who are less assertive may struggle to voice their opinions. It is also possible that participants in this study are more aware of disease risk information and genetic information because they were listed on a research register. A larger proportion of women vs. men participated in this study, and participants were also, on average, representative of an older demographic (mean age 56), which may limit the generalizability of our findings. However, genomic risk information could be used to encourage primary prevention, early detection and discussing risk with other family members; the latter two are particularly pertinent to older people.

On the basis of our findings and previous studies, when communicating genomic risk information with the aim of motivating healthy behaviours, we suggest the following:

1. Using the 100 person diagram risk format (especially for low literacy levels), and also consider presenting the double pie chart format;
2. Referring to absolute risk as ‘remaining lifetime risk’ rather than ‘lifetime risk’;
3. Including qualitative risk categories in addition to estimates of absolute and relative risk to help motivate appropriate behaviour change (although there may be some disadvantages to this approach^12^);
4. Providing the risk information to participants through a genetic counsellor or informed GP, either by telephone or face‐to‐face, in conjunction with written material delivered in a mode preferred by the participant.

The increasing role of genomic information in prediction of disease risk means we need to ask how and by whom this information should be provided. Understanding risk is vital to one of the key roles of public health genomics – to promote appropriate behaviour change to reduce risk of disease.

## Conflicts of interest

The authors have no conflict of interests.

## Sources of funding

This study was funded by a Sydney Catalyst Pilot and Seed Funding grant. AE Cust is supported by a NHMRC Career Development Fellowship (APP1063593) and a Cancer Institute NSW Early Career Fellowship (10ECF206).

## Supporting information


**Appendix S1**. Focus group discussion guide.
