# Supplementary material for: Public preferences for communicating personal genomic risk information: a focus group study
Source: Health Expect. 2015 Sep 1;19(6):1203–14. doi: 10.1111/hex.12406 (PMC5139046; doi:10.1111/hex.12406)
Supplement: Supplementary file 1 — Appendix S1. Focus group discussion guide. [file HEX-19-1203-s001.doc]

**APPENDIX 1 (SUPPLEMENTARY MATERIAL FOR ONLINE PUBLICATION ONLY)**

**Public preferences for communicating personal genomic risk information:**

**a focus group study**

**Focus Group DISCUSSION GUIDE**

**Script for focus group facilitator:** This will be spoken by the facilitator

Welcome to today’s Focus Group session!

My name is [facilitator’s name] and this is [other researcher], who are part of the [name of research team] with the [name of institute]. You have been invited to participate in today’s focus group session to help us develop an easy to understand method of talking about a person’s individual genetic risk of melanoma.

We would like to hear everyone’s ideas and feedback, and we encourage all of you to participate and contribute to the session. We do have quite a-lot to get through within the 2 hours so we ask for your understanding and cooperation, as we may sometimes have to interrupt discussions in order to move onto our next talking point. We are not looking for you to agree about anything so it is completely fine for you to disagree with each other, but obviously please try not to criticise others.

*[Explain that we are not doctors and cannot give medical advice but we will answer questions to the best of our ability and they can follow up with their own doctor.]*

*[Request participants to switch off mobile phones or put them on ‘silent’.]*

Our discussion today will have two parts. The first part will explore how people understand melanoma risk and different ways of communicating genetic risk as well as the potential impact of receiving information on personal genetic risk of melanoma for you and your family. We will use made-up examples only, you will not be given your own personal genetic risk of melanoma. We will then have a short break. Light refreshments, tea and coffee will be available during the break. The second part of the session will explore whether a genetic counsellor could help with the communication process.

We would like to make an audio recording of this session, just to make sure we can accurately summarize the discussion. Everything that’s said or written today will be kept strictly confidential. When the study results are reported, it will not be possible to identify any individual participants.

*[To discuss location of toilets, fire exits, practical issues]*

Are there any questions?

We will start off the session by getting you to fill out a short questionnaire about yourself. If anything is unclear in the questionnaire, please feel free to ask one of us to clarify for you.

**Description of Focus Group sessions**

| **THEMES** | **FLOW OF DISCUSSION AND KEY QUESTIONS** | ADDITIONAL PROMPTS | Estimated time |
| --- | --- | --- | --- |
| PART 1   - Risk Understanding - Risk Presentation - Ethical, Social and   Psychological implications | *Script above and distribute basic questionnaire to collect information about age, sex, skin colour, eye colour, hair colour, ethnicity, city/country of birth, sunburn, current sun protection behaviours (Recorded individually)*  *[With participants’ consent,* ***start recording****. Request that people speak up and avoid speaking at the same time as others.]*  *[Ask people to introduce themselves* ***briefly*** *– first names.]*  INTRODUCTION:  As you may be aware, melanoma is the most serious type of skin cancer in Australia and we are trying to improve its prevention and early diagnosis. We know that a person’s genetic make-up can contribute to their risk of getting melanoma, even when a person has no family history of melanoma. These genetic changes can be inherited and passed on to future generations. We now know quite a lot about the genetic changes that increase a person’s risk of melanoma, and we can test for some of these using a blood or saliva sample.  Currently genetic testing through the public health system is only available for a rare gene change that puts you at a high risk of developing a melanoma. However, as we find more common gene changes that contribute to the risk of developing melanoma, in future, we can expect testing to be available for these more common gene changes.  We are going to be talking a lot about melanoma risk, so, to clarify: in today’s session when we are talking about risk, we are talking about the chance of a person developing melanoma over their lifetime.  1) Before we begin, can you tell us what you already know about melanoma risk?  2) Does anyone know what the risk factors of melanoma are?  3) What do you think we mean when we say “genetic risk of melanoma”?  We will now look at a hypothetical risk scenario, and a few risk presentation formats. We would like you to tell us what the risk presentations mean to you, and whether or not they are easy to understand. We know people don't always think about risk in terms of numbers and graphs, and some people find graphs confusing, that is why we would like to know how you interpret these presentations of melanoma risk.  ***Distribute hypothetical scenarios with risk presentation formats (5 formats)***  Let’s look at the Scenario and Format 1 – *read out scenario and presentation format*  4) What does the graph mean to you?  5) What do you think Sarah’s risk is?  6) Do you have any comments on the way it is presented?  7) What do you like about this format? 8) What don’t you like about  this format? 9) Can you make suggestions for improving it?  ***Repeat questions for other formats (2, 3, 4, 5) – wording is the same***  **Can you please indicate on this form (show paper) your preferences for the different risk presentation formats, ordering 1 as your favourite to 5 as your least favourite.**  10) Would it matter if this information was presented online or on paper?  One of our research questions is whether this type of information would lead to people making better decisions about preventing melanoma, so we would like your views on this also.  11) How do you think people might respond to this information about their own genetic risk?  12) What concerns might people have about receiving this information?  13) Do you think it will influence how much time people spend in the sun or how they use sun protection?  14) Do you think people would be interested in knowing this information?  15) How do you think people could use this information?  16) Do you think anyone should be able to find out this information for themselves, or should it only be given by a health professional?  17) Do you think a person who gets this information would share it with their family? Why or why not? | Is it something you ever think about? Talk to others about?  Do you think there may be genetic or inherited risk factors?  Is it easy to understand?  Is there anything unclear?  How do you think people might respond to this information?  Do you think people would feel any different after receiving this information?  How might receiving this information make people feel? Anxious? Stressed?  Relieved? No Difference?  In what way?  Why or why not? If so why? At what ages/stage in life?  *Practical:* Sun protection behaviours  Skin examination behaviours  Time spent in the sun  *Psycho-social*: insurance, family communication  Would they feel comfortable?  Important for family to know? | 1 Hour |
| BREAK |  |  | 5-10 mins |
| PART 2  Genetic counsellor assistance | *Reanu: Briefly introduce the role of a genetic counsellor*  A Genetic counsellor is someone who is able to provide information to individuals and families about genetic conditions including certain types of cancer. They organize and assist with genetic testing and screening and deliver genetic test results and risk information about genetic conditions. They also provide options and support for the decision-making process and help with coming to terms with the impact of genetic test results and communicating this information within families.  18) If people were sent out information about their personal genetic risk of melanoma, do you think they would be interested in talking to a genetic counsellor about it?  19) Let’s think back to Sarah, whose risk we looked at earlier. Imagine that when she gets her genetic risk information, Sarah makes an appointment to see a genetic counsellor. What sorts of questions do you think she might want to ask the genetic counsellor?  20) Do you think it would be better to talk to a genetic counsellor or other health professional over the phone or in person?  21) Currently in Australia, through the health system, genetic risk information is only given to people with lots of cancers in the family, and  can only be given through a genetic counsellor. What do you think about this?  22) Is there anything about what we have discussed today that concerns or bothers you?  23) Is there anything else that you would like to say or ask that we haven’t already discussed today?  *THANK ALL PARTICIPANTS FOR THEIR CONTRIBUTION TODAY.* | Specific concerns?  Inheritance?  Potential concerns of passing on this genetic risk?  Genetic testing?  Concerns about sharing information with family? | 30 mins |

End of Session (1 Hour 45 mins)
